# Research trends and hotspots of metabolites and inflammatory bowel disease: a bibliometric analysis

**DOI:** 10.3389/fmicb.2025.1548233

**Published:** 2025-04-29

**Authors:** Jia-Qi Lyu, Fang-Jun Xiao, Ke-Ying Wang, Ying-Jie Liu, Jing-Mei Hui, Jie Lin

**Affiliations:** ^1^Shenzhen Hospital of Guangzhou University of Chinese Medicine (Futian), Shenzhen, Guangdong, China; ^2^Guangzhou University of Chinese Medicine, Guangzhou, Guangdong, China

**Keywords:** metabolites, inflammatory bowel disease, bibliometric, visual analysis, hotspots

## Abstract

**Purpose:**

The purpose of this study is to analyze the current research status and explore the relationship between metabolites and inflammatory bowel disease (IBD), providing insights for future research.

**Methods:**

In this study, we retrieved publications on metabolites and IBD from the Web of Science Core Collection (WOSCC), covering the period from 1994 to 2024. We conducted descriptive and visual analyses of the topics, journals, countries/regions, institutions, authors, and citation counts of these publications.

**Results:**

From January 1994 to June 2024, a total of 509 relevant publications were retrieved from the WOSCC, with the number of publications steadily increasing each year. These articles were published in 222 journals, with the top three most productive journals being inflammatory bowel diseases (36 publications), Alimentary Pharmacology & Therapeutics (16 publications), and Digestive Diseases and Sciences (13 publications). The leading countries in publication output were China (154 publications, 30.3%), the USA (101 publications, 19.8%), and the UK (32 publications, 6.3%), with total citation counts of 3,175, 7,439, and 1,444, respectively. The most recent trending keywords in this field include “gut microbiota,” “inflammation,” and “pathogenesis.”

**Conclusion:**

Recent research on the relationship between metabolites and inflammatory bowel disease (IBD) has grown significantly, deepening our understanding of their connection. Further exploration of this relationship could not only enhance the quality of life for IBD patients but also offer new insights into potential cures for the disease.

## 1 Introduction

Inflammatory bowel disease (IBD) is a group of chronic gastrointestinal disorders, including Crohn’s disease and ulcerative colitis, characterized by persistent intestinal inflammation. The course of IBD is unpredictable, with alternating periods of acute flare-ups and remission (Graham and Xavier, 2020; Kobayashi et al., 2020). The incidence of these diseases has increased significantly worldwide, particularly in developed countries and urban areas, posing a significant challenge to patients’ quality of life and placing a heavy burden on healthcare systems (Ng et al., 2017; Coward et al., 2019; Jones et al., 2019). Although IBD is currently incurable, it can be managed through treatments aimed at alleviating symptoms and improving quality of life (Eisenstein, 2018). The etiology of IBD is complex, involving genetic, environmental, and immune factors (Bruner et al., 2023). Common symptoms include persistent abdominal pain, diarrhea (with or without blood), fatigue, and weight loss. Extraintestinal manifestations (EIMs) such as aphthous stomatitis, iritis, episcleritis, uveitis, arthritis, and cardiovascular diseases (CVDs) are also common (Lichtenstein et al., 2018; Rubin et al., 2019). These symptoms can severely impact patients’ daily lives and mental health and may appear even before diagnosis (Bruner et al., 2023). Therefore, in-depth research into the pathogenesis of IBD and the development of effective treatment strategies are essential for improving patient prognosis and quality of life.

Metabolites are small molecules produced as intermediates or end products of microbial metabolism, originating from either bacterial metabolism, host molecules, or directly from bacteria (Lavelle and Sokol, 2020). In recent years, metabolite research has gained increasing attention, particularly in the context of chronic diseases and metabolic syndromes. Metabolites not only reflect the body’s health status but also serve as potential biomarkers, offering crucial information for early diagnosis and disease monitoring (Li et al., 2022). By studying metabolite changes, we can gain deeper insights into the role of the gut microbiota and its impact on host health, helping to explore the biological mechanisms behind IBD. The link between metabolites and IBD has garnered growing interest. Studies have shown that gut microbiota-derived metabolites, such as short-chain fatty acids, significantly influence intestinal immunity and barrier function (Adolph et al., 2022). Abnormal changes in these metabolites are closely associated with the pathological progression of IBD, highlighting their potential as biomarkers and therapeutic targets. While metabolomics analyses have identified 36 significantly altered metabolites, their precise roles in IBD remain unclear. Therefore, investigating the relationship between metabolites and IBD is crucial, as it not only enhances our understanding of IBD pathogenesis but also aids in developing novel therapeutic strategies. Bibliometric analysis, a statistical method that retrospects data, identifies correlations, and forecasts future developments, has become a key tool in studying research trends (Thompson and Walker, 2015). Visualization tools such as CiteSpace and VOSviewer are widely used to track and analyze the evolution of research trends and emerging hotspots in a specific field (Cheng et al., 2021). In recent years, several bibliometric studies have explored topics such as gut microbiota and IBD (Xu et al., 2022) and metabolomics in gastrointestinal diseases (Zhang et al., 2023). However, none of these analyses have specifically focused on the relationship between metabolites and IBD, a rapidly evolving area at the intersection of immunology, microbiology, and metabolomics. Therefore, our study aims to fill this gap by conducting a comprehensive bibliometric analysis to reveal research trends and hotspots in this unique domain.

## 2 Materials and methods

### 2.1 Literature sources and search strategies

**Figure 1:**
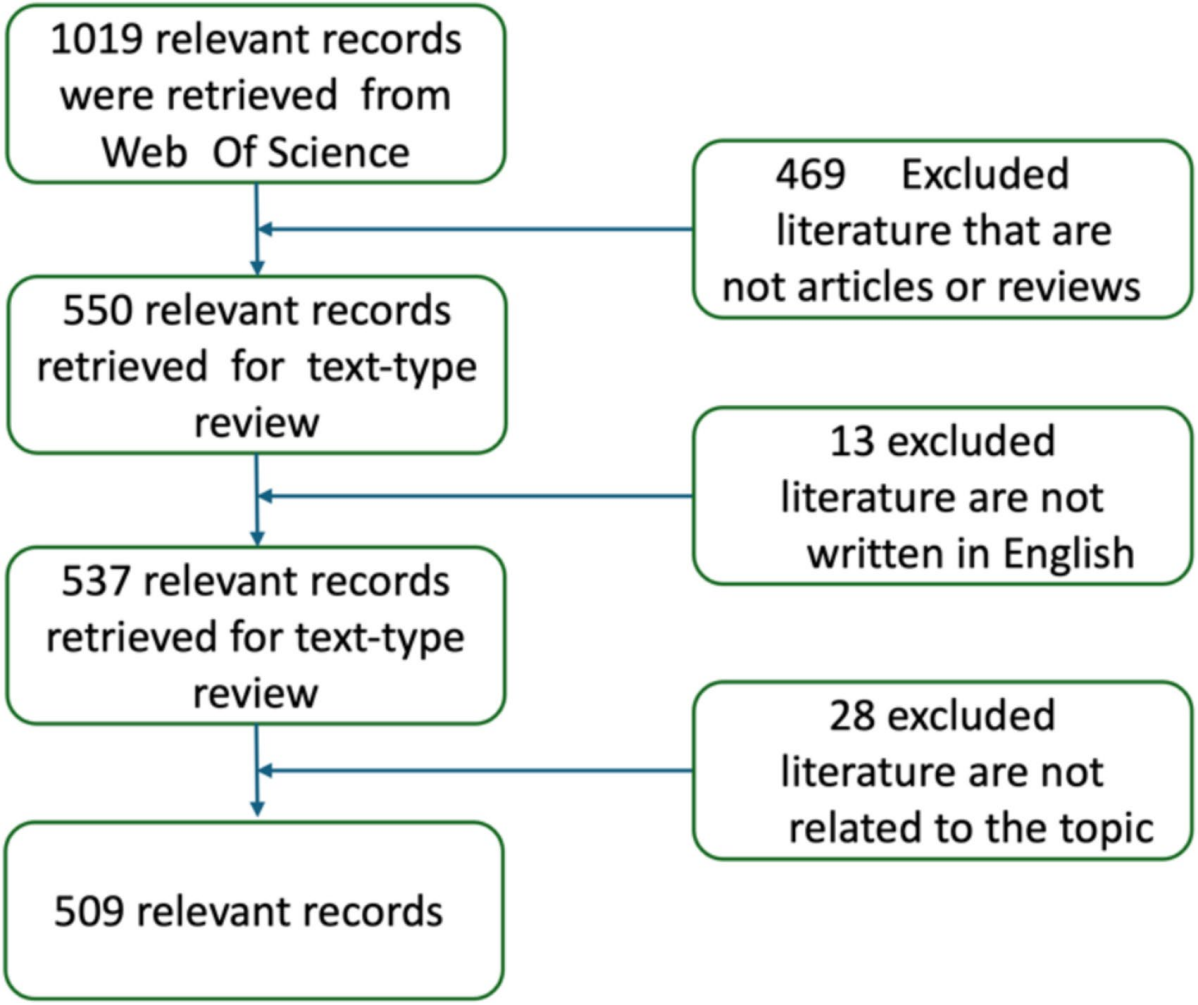
Flow diagram of research selection and screening.

The articles selected for this study were sourced from the WOSCC. The WOSCC is a highly authoritative database that covers the most majority of SCI-indexed literature. This platform was chosen for its comprehensiveness and authority, as it includes a vast number of high-quality, peer-reviewed journal articles. Additionally, it provides detailed citation information and bibliometric analysis tools, which are essential for systematically assessing research trends and identifying emerging hotspots. Web of Science’s frequent updates and advanced search functionalities make it particularly suitable for interdisciplinary research, offering reliable support for analyzing metabolites and research trends and hotspots in inflammatory bowel disease (Yin et al., 2021; Zhang et al., 2024). To ensure the accuracy and quality of the retrieved data, indexes such as SCI-Expanded, CCRI-Expanded, and IC were selected. The search strategy used was as follows: ((TI = (inflammatory bowel disease)) OR TI = (ulcerative colitis)) OR TI = (Crohn’s disease) AND (((TI = (Metabolomics)) OR TI = (Metabolism)) OR TI = (Metabolite)) OR TI = (Metabolic). Both subject terms and free-text keywords were employed during the retrieval process. The retrieved literature was then screened, with the specific process outlined in Figure 1. A total of 1,019 relevant records were obtained from Web of Science, covering the period from January 1, 1994, to June 30, 2024. The data collected included titles, keywords, publication dates, authors, institutions, countries or regions, journals, and total citations. To perfect the research and minimize bias caused by frequent database updates, all literature searches and data downloads were completed within a single day, on October 24, 2024, and two independent researchers handled irrelevant and duplicate literature, respectively. Any different opinions were discussed until consensus was reached. The Web of Science database is the sole source of information for this study, and since no human subjects were involved, ethical informed consent was not required.

### 2.2 Inclusion and exclusion criteria of literature

Inclusion Criteria: (1) The literature must be original research articles or review papers. (2) Publications must be written in English. (3) The subject matter must directly pertain to metabolites and IBD. Exclusion Criteria: (1) Documents of other types, such as meeting abstracts, editorials, or letters. (2) Non-English publications. (3) Republished studies. (4) Materials not directly relevant to the research topic.

### 2.3 Research methods

Data analysis and literature visualization were performed using CiteSpace 6.3.1 and VOSviewer 1.6.20 and R4.4.1. CiteSpace, developed by Professor Chen Chaomei, is a tool designed to visualize collaborative networks, citation networks, and research hotspots (Chen, 2004; Chen, 2006). VOSviewer, created by the Scientific and Technological Research Center of Leiden University, specializes in visualizing bibliometric networks. Both tools provide objective and varied perspectives for comprehensive data analysis. Using these bibliometric tools, we examined trends in annual publication and citation counts across different countries and regions. Keywords extracted from the selected literature were grouped into distinct clusters via co-occurrence analysis in VOSviewer, with each cluster color-coded based on its temporal occurrence. Additionally, CiteSpace was utilized for analyzing collaborations among institutions and journals, as well as for co-occurrence analysis of keywords. The software also identified significant citation bursts for institutions, journals, and keywords. Furthermore, Bibliometrix was used to visualize the number of publications per country and the collaborative relationships between countries.

## 3 Results

### 3.1 Trends in global publication volume

Using the outlined search strategy, we initially retrieved 1,019 papers. After excluding 469 papers that were not articles or reviews, 550 papers remained. We then removed 13 non-English papers. Further screening of titles and abstracts led to the exclusion of 28 papers unrelated to our study’s focus. Finally, after reviewing the full texts, 509 papers were included in this bibliometric analysis. The selection process is shown in Figure 1.

**Figure 2:**
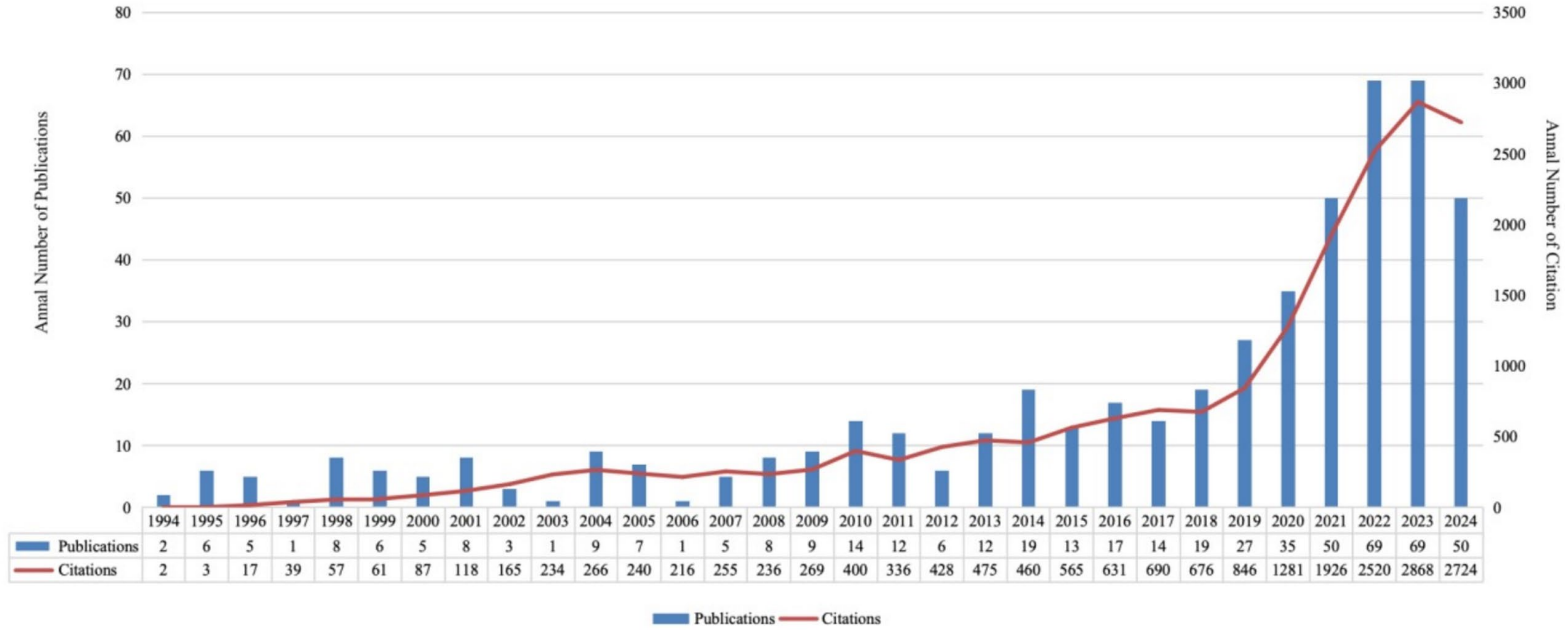
Annual publications and citations in metabolites and inflammatory bowel disease from 1994 to 2024.

This study included 509 papers authored by 3,487 researchers from 1,039 institutions across 52 countries. These papers were published in 222 different journals and cited a total of 12,456 sources from 1,971 journals. From 1994 to 2024, the number of publications in the fields of metabolites and IBD showed a consistent upward trend. Notably, the volume of publications began to increase significantly in 2019, with a sharp surge in 2022, as shown in Figure 2.

### 3.2 Author distribution

Table 1 shows the top five prolific authors, each of whom has published more than six articles, with all of their articles being cited more than five times. Umberto Cillo is the most prolific author in this field, with seven publications and 307 citations, averaging 43.86 citations per year. In contrast, Curtis Huttenhower holds the highest citation count, with a total of 1,315 citations and an average of 219.17 citations per year. Together, these two authors have made the most significant contributions to the research on metabolites and IBD.

**Table 1:** Top five authors and citation frequency of literature related to metabolites and IBD in WOS database.

Rank	Corresponding authors	Number of studies	Total citations	Average per year	H-index
1	Cillo, Umberto	7	307	43.86	7
2	Huttenhower, Curtis	6	1,315	219.17	5
3	Mingrone, Geltrude	6	229	38.17	6
4	van Bodegraven, Adriaan A.	6	83	13.83	5
5	Capristo, Esmeralda	6	229	38.17	6

### 3.3 Analysis of publishing journals

The top ten journals with the highest number of publications on metabolites and IBD published a total of 140 articles, accounting for 27.5% of the total literature (Table 2). To assess the academic influence of these journals, we referred to the 2023 Journal Citation Reports (JCR) to obtain the latest impact factor (IF) (Eyre-Walker and Stoletzki, 2013). The top five journals are: Inflammatory Bowel Diseases (36 articles, 7.1%), Alimentary Pharmacology & Therapeutics (16 articles, 3.1%), Digestive Diseases and Sciences (13 articles, 2.6%), International Journal of Molecular Sciences (12 articles, 2.4%), and Scientific Reports (12 articles, 2.4%).

**Table 2:** Top ten productive journals related to metabolites and IBD.

Rank	Journal	Studies counts	Total citations	Average per year	IF in 2023
1	Inflammatory Bowel Diseases	36	1,150	31	4.50
2	Alimentary Pharmacology & Therapeutics	16	796	49	6.60
3	Digestive Diseases and Sciences	13	364	28	2.50
4	International Journal of Molecular Sciences	12	219	18	4.90
5	Scientific Reports	12	180	15	3.80
6	Journal of Ethnopharmacology	11	152	13	4.80
7	Nutrients	11	277	25	4.80
8	Journal of Crohns & Colitis	10	246	24	8.30
9	World Journal of Gastroenterology	10	274	27	4.30
10	Gastroenterology	9	2,448	272	25.70

**Figure 3:**
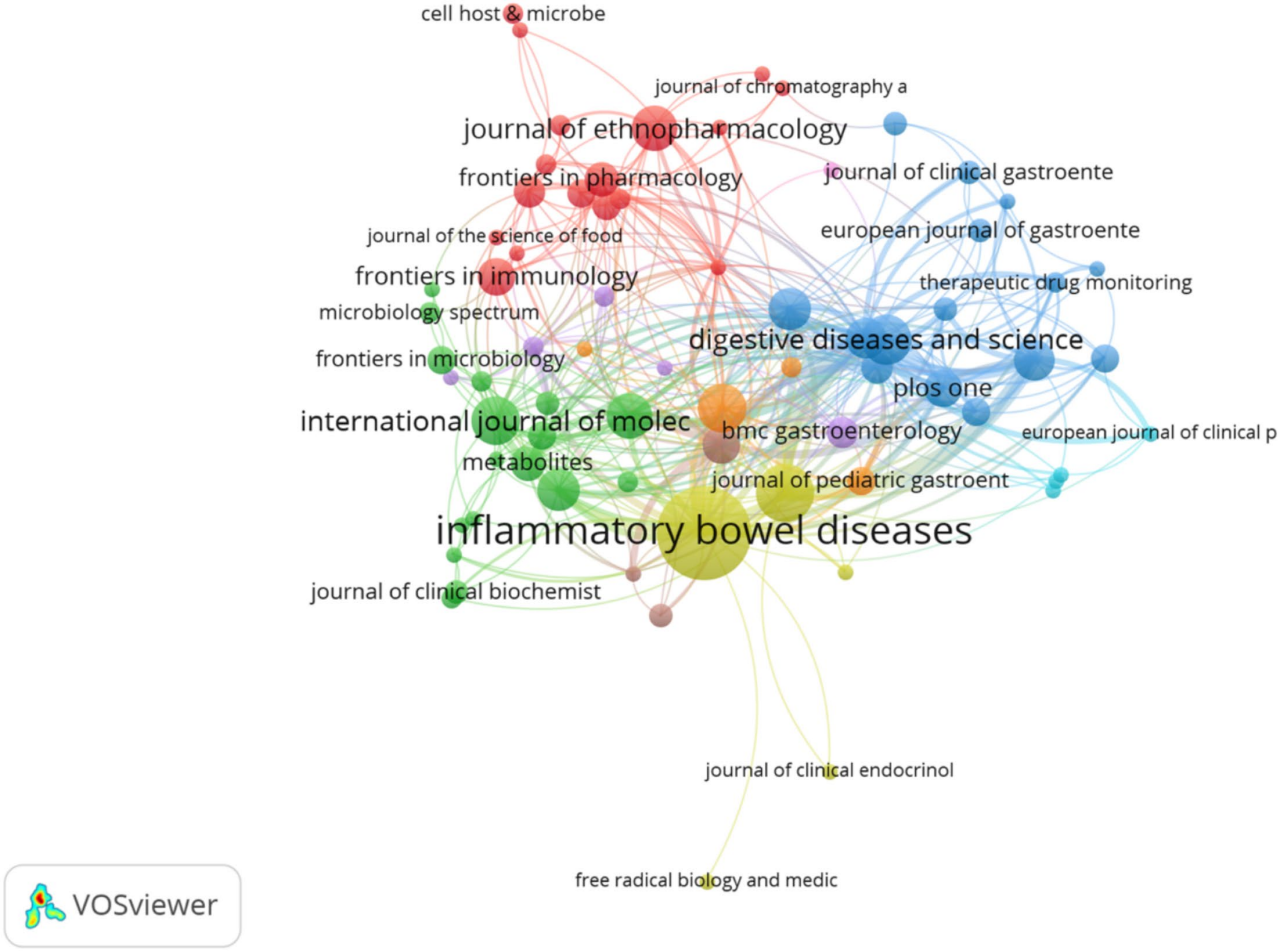
Citation analysis of published journals.

Figure 3 presents a co-citation analysis of published journals conducted using VOSviewer, where the size of each node represents the number of citations. The analysis reveals that Inflammatory Bowel Diseases, Journal of Ethnopharmacology and Digestive Diseases and Sciences are highly cited, positioning them at the center of the co-citation network. Additionally, although Gastroenterology has a smaller number of articles published, it is the most frequently cited journal, with an impressive IF of 25.70.

### 3.4 Analysis of communications from different countries

In terms of the number of published papers, China ranked first with 154 articles (30.3%), followed by the USA with 101 articles (19.8%), and England with 32 articles (6.0%). Japan (32 articles, 6.0%), Canada (30 articles, 5.9%), and the Netherlands (26 articles, 5.1%) also contributed significantly to the field. Table 3 shows the six countries with the highest publication counts. Regarding total citation volume, the USA leads with 7,439 citations, followed by China with 3,175, Canada with 2,339, the Netherlands with 1,928, and England with 1,444. While China has the largest number of publications, the USA has the highest citation count.

**Table 3:** Top six countries and citation frequency of metabolites and IBD literature published in WOS database.

Rank	Countries	Studies counts	Total citations	Average per study
1	China*	154	3,175	20
2	USA	101	7,439	73
3	England	32	1,444	45
4	Japan	32	482	15
5	Canada	30	2,339	77
6	Netherlands	26	1928	74

^*^Taiwan and China use the same codes.

**Figure 4:**
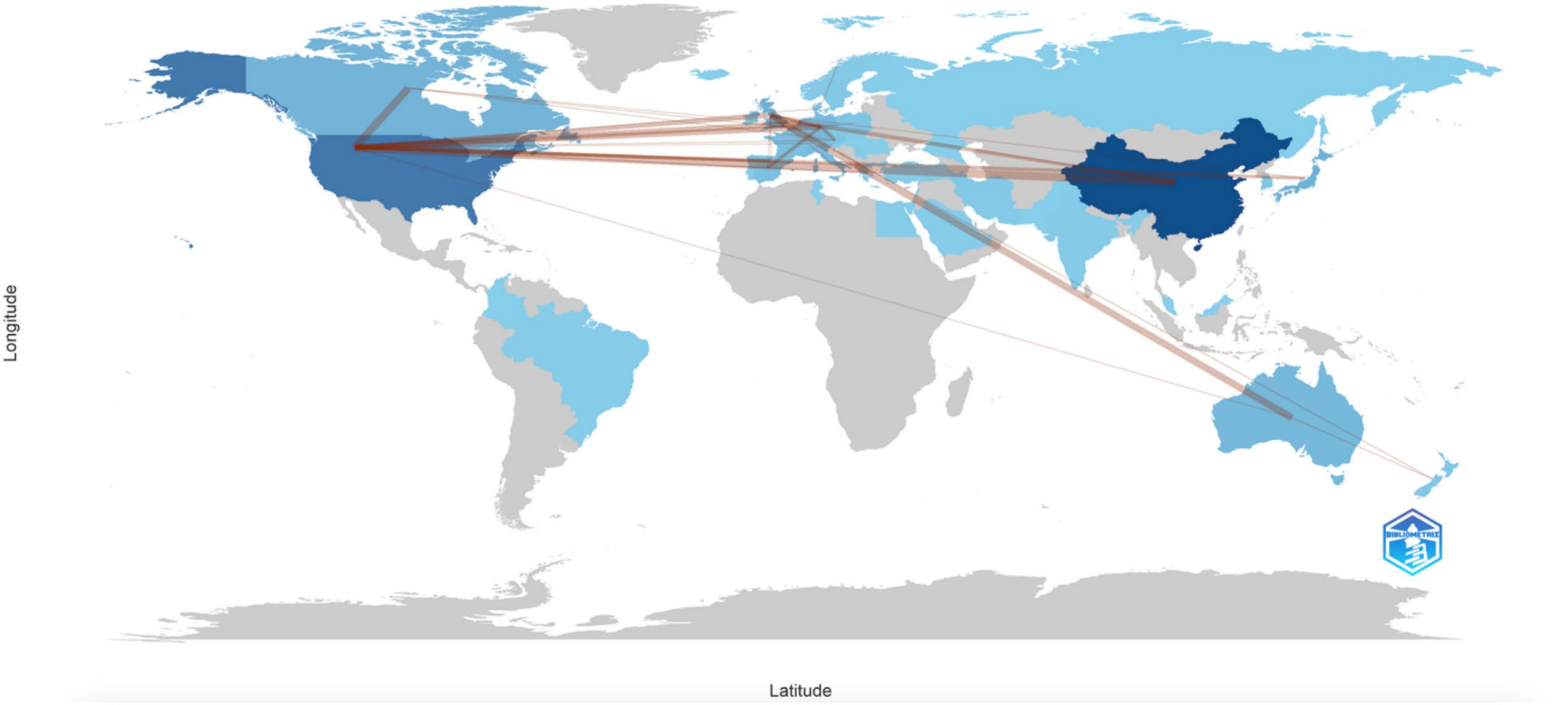
Countries collaboration analysis. The lines represent connections between countries. The color intensity of each country on the map corresponds to the number of publications, with darker colors indicating more publications. The number of connecting lines reflects the level of collaboration between countries.

We analyzed the level of collaboration between countries in the field of metabolites and IBD and visualized these relationships using R, as shown in Figure 4. The color intensity indicates the number of publications, while the connections between countries represent direct collaborative relationships. Notably, China leads in the number of publications and maintains close collaboration with the US and the UK. Meanwhile, the USA has the most extensive international research partnerships, particularly with Canada, China, the UK, and several European countries.

### 3.5 Analysis of article publishing organizations

**Figure 5:**
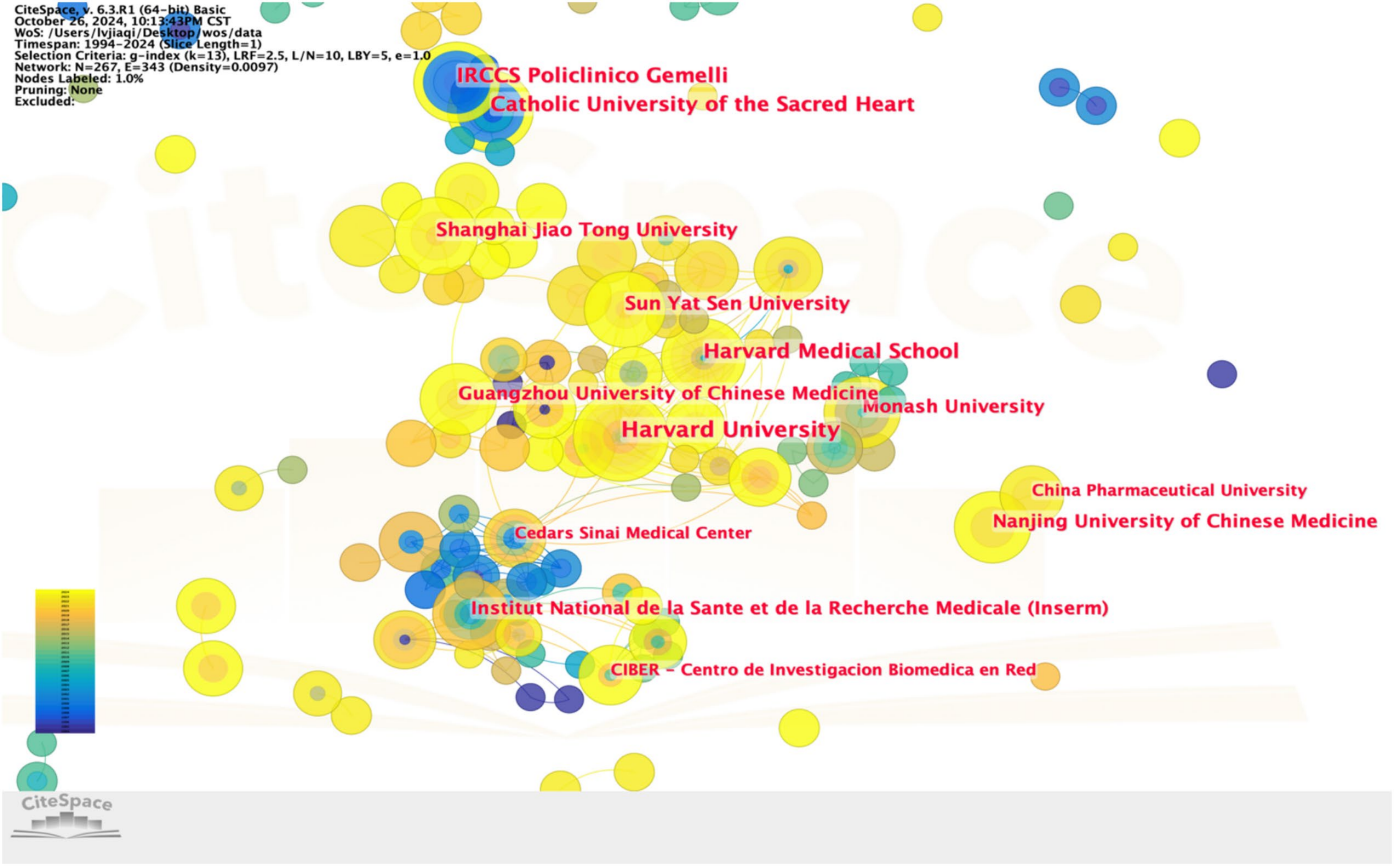
Global institution collaboration analysis: the nodes represent institutions, and the lines represent connections between them. The size of each node is proportional to the number of publications, while the thickness of the lines indicates the degree of collaboration between institutions. From 1994 to 2024, the color gradually changes from dark blue to light yellow.

On a global scale, Harvard University and the University of California System (14 articles, 2.75%) lead in article output over the past 30 years in this field. Their publications have accumulated 123 and 143 citations, respectively. Among the top ten ranked institutions, three are from the USA, three from China, and the remaining four include the Institut National de la Santé et de la Recherche Médicale (France), Vrije Universiteit Amsterdam (Netherlands), and Catholic University of the Sacred Heart and IRCCS Policlinico Gemelli (Italy) (Table 4). Subsequently, we used CiteSpace to perform a visual analysis of interagency collaboration, highlighting the patterns of cooperation between institutions (Figure 5).

**Table 4:** Top ten institutions published studies related to metabolites and IBD.

Rank	Institutions	Country	Studies counts	Total citations	Average per year	Percentage (N/509)
1	Harvard University	USA	14	1729	123	2.75
2	University of California System	USA	14	2005	143	2.75
3	Institut National de la Sante et de la Recherche Medicale	France	12	1,412	117	2.35
4	Sun Yat Sen University	China	11	93	8	2.16
5	Harvard Medical School	USA	10	1,471	147	1.96
6	ShangHai Jiao Tong University	China	10	126	12	1.96
7	Vrije Universiteit Amsterdam	Holland	10	208	20	1.96
8	Catholic University of the Sacred Heart	Italy	9	310	34	1.76
9	IRCCS Policlinico Gemelli	Italy	9	310	34	1.76
10	Nanjing University of Chinese Medicine	China	9	107	11	1.76

### 3.6 Analysis of keywords of the publications

**Figure 6:**
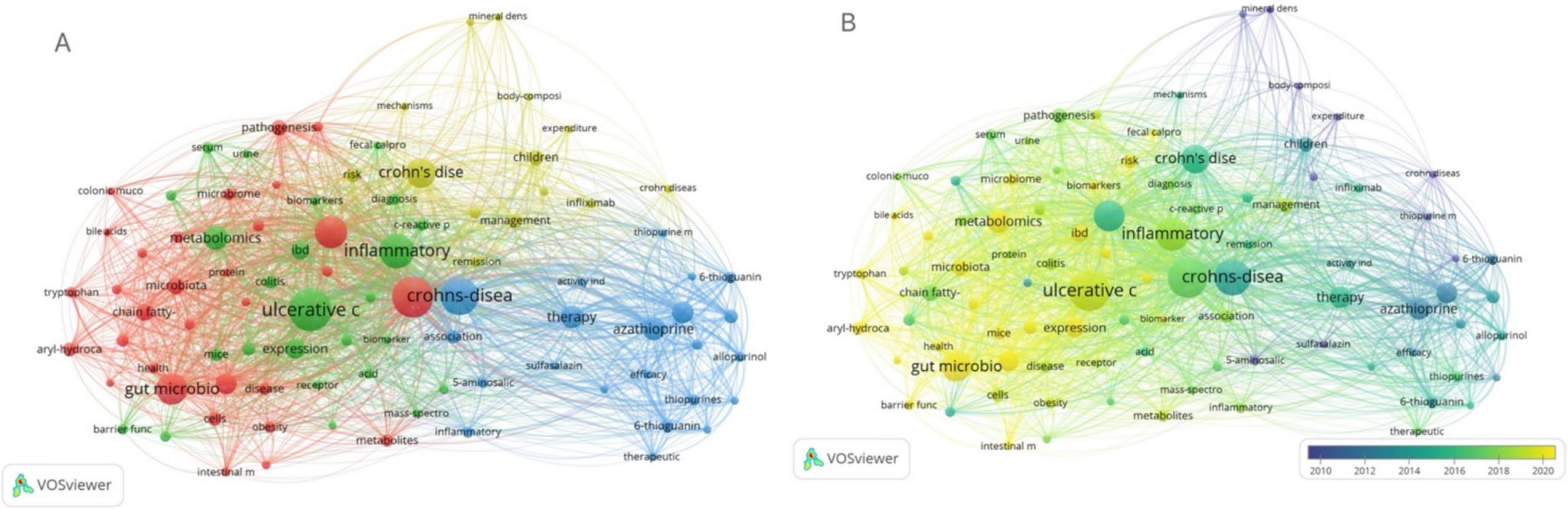
A VOSviewer network visualization of keywords in metabolites and IBD publications. **(A)** Mapping of the keywords in the area. Based on the default color scheme, the keywords are divided into four clusters: research related to gut metabolites (red), research related to inflammatory bowel disease treatment (blue), research related to ulcerative colitis (green), and research related to Crohn’s disease (yellow). Larger circles represent keywords with higher frequency of occurrence. **(B)** The distribution of keywords is presented according to their average appearance time. High-frequency keywords are primarily concentrated between 2010 and 2020. Blue represents earlier appearances, while yellow indicates more recent appearances. The smaller the distance between two keywords, the greater the frequency of their co-occurrence.

From the titles and abstracts of the 509 included articles, 92 keywords with a frequency of 10 or more were extracted, and a co-occurrence analysis of these keywords was performed using VOSviewer. In Figure 6A, the 92 keywords are grouped into four clusters: Cluster 1 (studies related to gut metabolites, red), Cluster 2 (studies related to the treatment of IBD, blue), Cluster 3 (studies related to ulcerative colitis, green), and Cluster 4 (studies related to Crohn’s disease, yellow). The size of each node represents the frequency of keyword occurrences. The nodes are color-coded based on the time period when the keywords were first introduced, as shown in Figure 6B. High-frequency keywords primarily emerged between 2010 and 2020, with dark blue indicating earlier keywords and yellow representing more recent ones.

**Figure 7:**
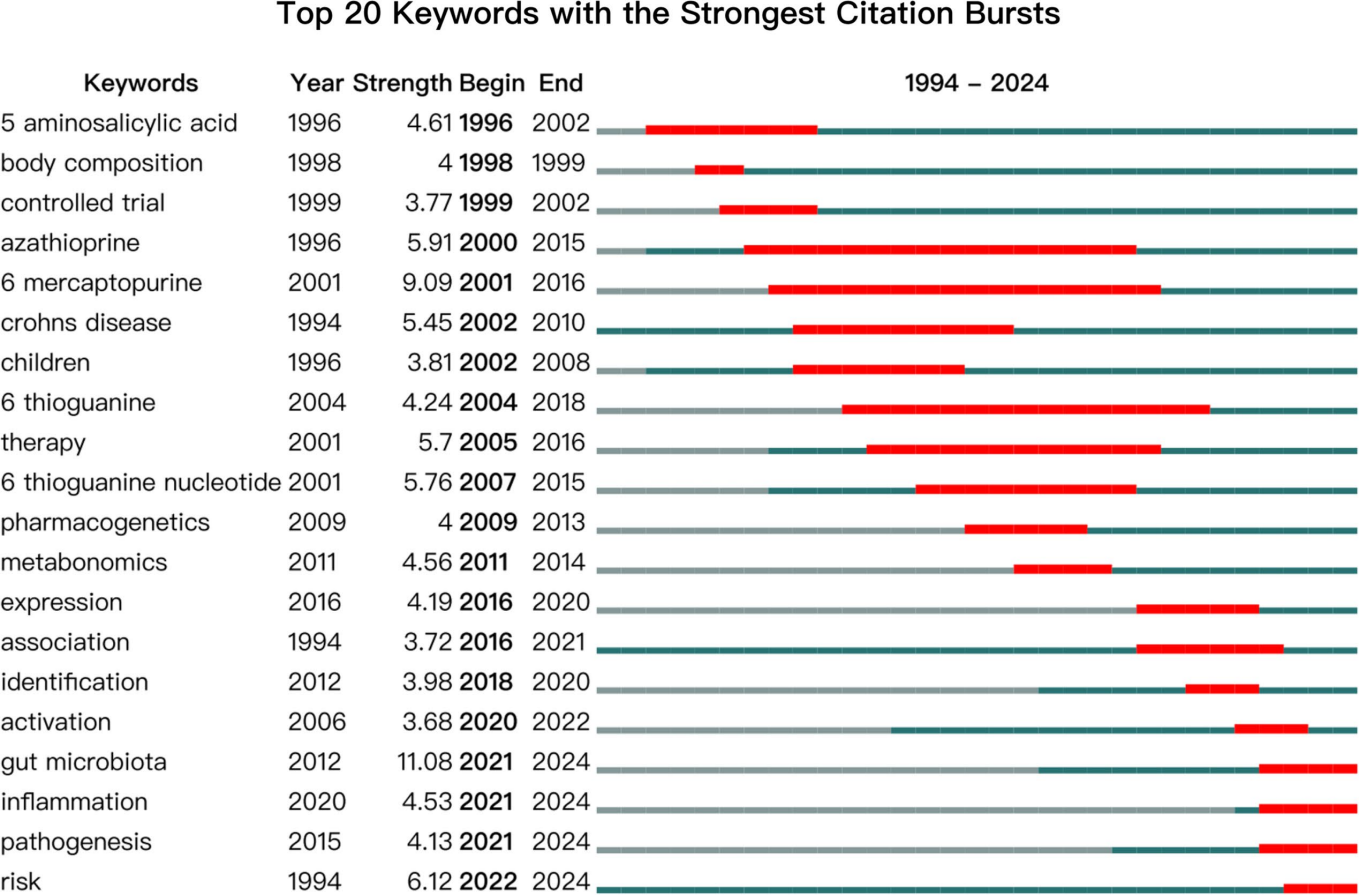
Top 20 keywords with the strongest citation bursts.

To further explore research hotspots in the field of metabolites and IBD, we conducted a burst analysis using CiteSpace on articles published between 1994 and 2024, identifying 20 burst keywords. Burst keywords are those that appear frequently within a specific time frame, reflecting the evolution of research trends and offering insights into emerging topics and future directions. As shown in Figure 7, “azathioprine” (burst intensity 5.91), “6-mercaptopurine” (burst intensity 9.09), and “6-thioguanine” (burst intensity 4.24) were prominent topics from 2004 to 2014. In the subsequent decade, “gut microbiota” (burst intensity 11.08), “inflammation” (burst intensity 4.53), and “pathogenesis” (burst intensity 4.13) emerged as key areas of focus in research related to metabolites and IBD.

### 3.7 Analysis of the co-citations

**Figure 8:**
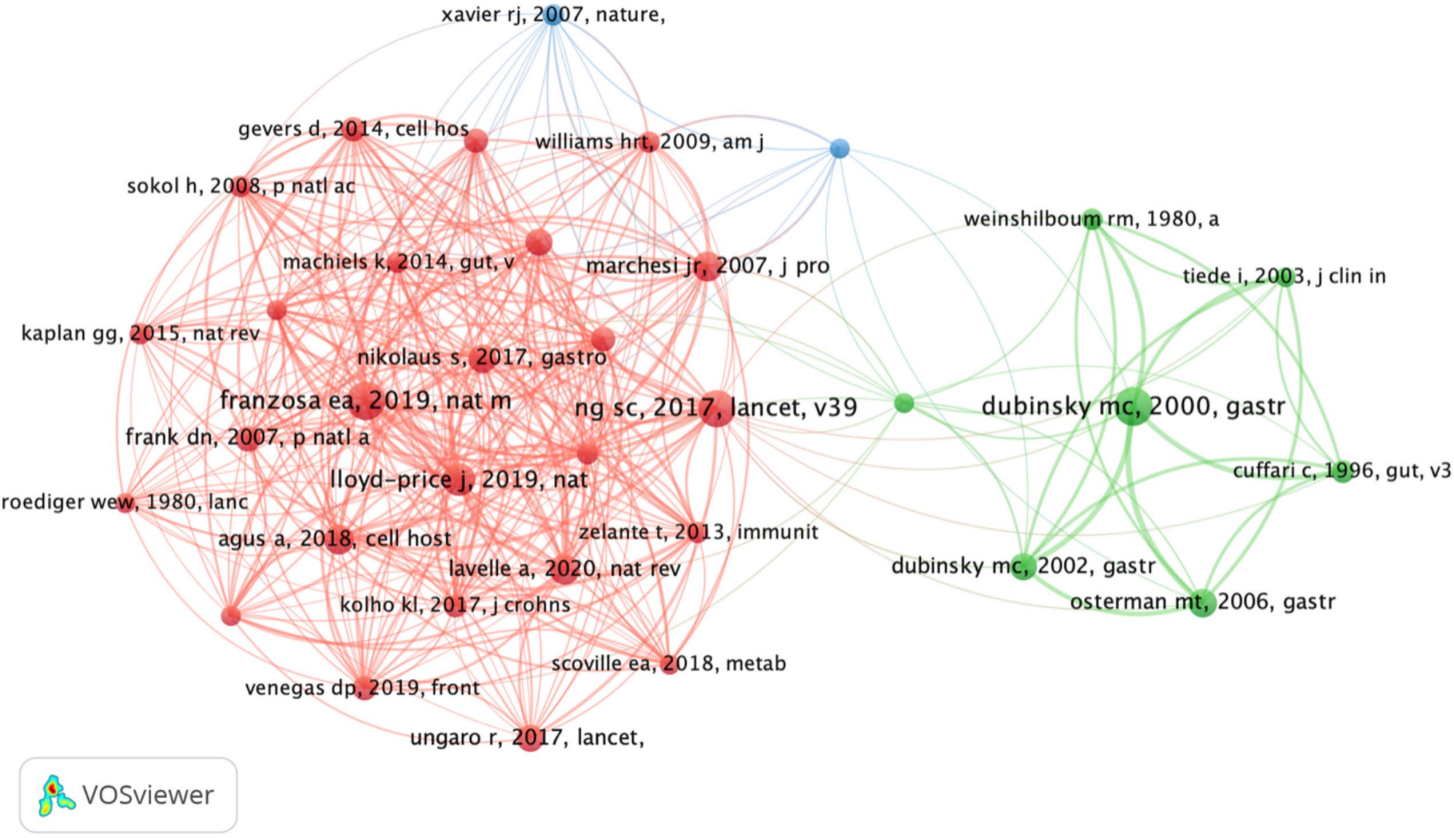
Co-citation map related to metabolites and IBD.

We ranked the 509 retrieved articles by citation frequency and listed the top 10 most cited articles (Table 5). The most cited article is Gut Microbiome Structure and Metabolic Activity in Inflammatory Bowel Disease (Franzosa et al., 2019), authored by Curtis Huttenhower and Ramnik J. Xavier, published in 2019 in Nature Microbiology. This article has garnered 1,055 citations, with an average of 175.83 citations per year. Another highly cited article is Gut Microbiota-Derived Metabolites as Key Actors in Inflammatory bowel disease, published by Harry Soko et al. in 2020 in Nature Reviews Gastroenterology & Hepatology, which has accumulated 1,004 citations and an average of 200.8 citations per year. Additionally, we conducted a co-citation analysis using VOSviewer, which revealed a co-citation pattern for 34 articles that had been cited more than 20 times. The article Gut Microbiome Structure and Metabolic Activity in inflammatory bowel disease remains at the center of this co-citation network (Figure 8).

**Table 5:** Top ten high-cited papers related to metabolites and IBD.

Rank	Title	Corresponding authors	Journal	Publication year	Total citations	Average per year
1	Gut microbiome structure and metabolic activity in inflammatory bowel disease	Curtis Huttenhower, Ramnik J. Xavier	Nature Microbiology	2019	1,055	175.83
2	Gut microbiota-derived metabolites as key actors in inflammatory bowel disease	Harry Soko	Nature Reviews Gastroenterology & Hepatology	2020	1,004	200.8
3	Pharmacogenomics and metabolite measurement for 6-mercaptopurine therapy in inflammatory bowel disease	Ernest G. Seidman	Gastroenterology	2000	806	32.24
4	Microbiota metabolite short chain fatty acids, GPCR, and inflammatory bowel diseases	Yingzi Cong	Journal of Gastroenterology	2017	635	79.38
5	Metabolomics Reveals Metabolic Biomarkers of Crohn’s Disease	Philippe Schmitt-Kopplin	Plos One	2009	411	25.69
6	6-MP metabolite profiles provide a biochemical explanation for 6-MP resistance in patients with inflammatory bowel disease	Marla C. Dubinsky, Eric A. Vasiliauskas	Gastroenterology	2002	400	17.39
7	Increased tryptophan metabolism is associated with activity of inflammatory bowel diseases	Stefan Schreiber	Gastroenterology	2017	350	43.75
8	Role of reactive metabolites of oxygen and nitrogen in inflammatory bowel disease	Matthew B. Grisham	Free Radical Biology And Medicine	2002	327	14.22
9	Specific Bacteria and Metabolites Associated With Response to Fecal Microbiota Transplantation in Patients With Ulcerative Colitis	Nadeem O. Kaakoush	Gastroenterology	2019	305	50.83
10	6-Mercaptopurine metabolism in Crohn’s disease: Correlation with efficacy and toxicity	G. Seidman	Gut	1996	281	9.69

## 4 Discussion

Based on the WOSCC, we retrieved literature on metabolites and IBD published up to June 2024 and conducted a bibliometric visualization analysis. The goal of this study is to provide a comprehensive overview of the current research landscape, identify emerging research hotspots, track disciplinary trends, and offer insights for future directions. By analyzing research output and citation patterns, we aim to highlight the academic influence of countries, institutions, and scholars, shedding light on their contributions to this field.

The number of publications on metabolites and IBD has steadily increased over the years, with a significant peak in 2021, reflecting the growing importance of this research area. Several factors may explain this trend: (1) The rising global prevalence of IBD and the growing recognition of the link between gut microbiota and IBD via metabolites (Ning et al., 2023). (2) The increasing interest in metabolomics as a tool for advancing IBD treatment (Li et al., 2022). (3) Studies suggesting that IBD often requires long-term immunosuppressive therapy, with susceptibility to COVID-19 closely tied to immune function. Many experts now view these two issues as interconnected, driving concurrent research (Viganò et al., 2021). (4) The COVID-19 pandemic, which led to widespread lockdowns and increased isolation, provided researchers with more time to focus on advancing studies.

In terms of citation frequency and IF, Gastroenterology (IF = 25.70), Alimentary Pharmacology & Therapeutics (IF = 6.60), and Inflammatory Bowel Diseases (IF = 4.50) are key journals publishing articles on metabolites and IBD. Researchers in this field are encouraged to focus on literature from these high-impact journals to stay updated on the latest trends and advancements in international research. By doing so, they can broaden the scope and depth of their own work, thereby enhancing their global recognition and contribution to the field.

The number of publications in the field of metabolites and IBD from countries such as China, the USA, and England ranks among the highest. Both the U.S. and China lead in citation frequency, highlighting their significant academic influence. China, in particular, has published the most papers, contributing 30.3% of the total publications in this area. As global collaboration continues to grow, future research in this field is likely to see increasing cooperation between researchers from multiple countries.

Highly cited papers have a significant academic impact on research in a particular field. The top ten most cited papers in the area of metabolites and IBD are listed in Table 5. The most frequently cited study is Gut Microbiome Structure and Metabolic Activity in Inflammatory Bowel Disease, published in Nature Microbiology in 2019 (IF = 20.5). This article provides an overview of the research progress on the gut metabolome and microbiome in IBD. It evaluates the metabolic characteristics of IBD patients and explores the relationship between the gut metabolome, microbiome, and intestinal inflammation. The study identifies significant differences in metabolites in IBD patients, such as an enrichment of sphingolipids and bile acids, and a reduction in triglycerides and porphyrins. Metagenomic analysis reveals differentially abundant microbial species and functions associated with adaptive responses to oxidative stress. By integrating metabolomics and metagenomics data, the study uncovers potential mechanistic relationships, highlighting the diagnostic and therapeutic value of these features for IBD.

The latest burst keywords in the field of metabolites and IBD include “gut microbiota,” “inflammation,” and “pathogenesis.” The emergence of “gut microbiota” as a trending keyword underscores the increasing recognition of the gut microbial community as a key regulator of host metabolic and immune homeostasis. Recent studies have focused on microbiome-derived metabolites—such as short-chain fatty acids (Parada Venegas et al., 2019), indole derivatives (Li et al., 2024), and bile acids (Pan et al., 2024)—that influence epithelial integrity and immune signaling pathways, thereby contributing to the development and modulation of IBD. The keyword “inflammation” reflects a core pathological feature of IBD. The growing attention to metabolic regulators of inflammation highlights a mechanistic turn in the literature, wherein researchers are investigating how metabolic shifts in IBD patients drive dysregulated immune responses. This includes the study of metabolic pathways like the kynurenine pathway (Lai et al., 2023), arachidonic acid metabolism (Mayr et al., 2020), and sphingolipid signaling (Brown et al., 2019) in the context of intestinal inflammation. Finally, the keyword “pathogenesis” indicates an increasing focus on identifying the causal links between metabolic alterations and disease mechanisms. This trend suggests a move toward translational research aimed at discovering biomarkers and therapeutic targets (Qiu et al., 2023), offering potential for precision medicine approaches in IBD management. These emerging topics collectively demonstrate a paradigm shift in IBD research from observational associations to in-depth mechanistic exploration of host–microbe–metabolite interactions.

The evaluation of IBD involves several key clinical activity indices. The Harvey-Bradshaw Index (HBI) is commonly used for assessing Crohn’s disease, while the Simple Clinical Colitis Activity Index (SCCAI) is employed to monitor ulcerative colitis. These indices provide valuable insights into disease severity and patient outcomes. In addition to these activity scores, various biomarkers are crucial in the diagnosis and management of IBD. Many gut-specific biomarkers such as anti-*Saccharomyces cerevisiae* antibodies (ASCA), perinuclear anti-neutrophil cytoplasmic antibodies (pANCA), and fecal calprotectin levels are helpful for distinguishing IBD from other gastrointestinal diseases and for monitoring disease activity and response to treatment. These biomarkers not only aid in diagnosis but also in predicting disease flare-ups and guiding therapeutic interventions. The increasing understanding of gut microbiota and its metabolites as integral players in IBD pathogenesis offers promising new directions for treatment strategies aimed at modulating the microbiome and its metabolic products. Many IBD symptoms are closely linked to the metabolites produced by the gut microbiota. These metabolites play a crucial role in maintaining intestinal homeostasis (Deleu et al., 2021). Butyrate is a metabolite closely associated with IBD. It is produced by *F. prausnitzii* and exerts anti-inflammatory effects by inhibiting the IL-6/STAT3/IL-17 signaling pathway and promoting Foxp3 expression. A decrease in butyrate levels may induce the onset of colitis (Matsuoka and Kanai, 2015; Yu et al., 2024). Additionally, bacteriocins serve as important antimicrobial agents (Zhou et al., 2018). Lactobacillus species in humans produce lactobacillin, which inhibits *Listeria monocytogenes* infection (Rolhion et al., 2019). Current research shows significant alterations in the gut microbiota of IBD patients. Notably, several butyrate-producing bacteria, including *Faecalibacterium prausnitzii*, *Roseburia* spp., *Eubacterium hallii*, *Anaerobutyricum* spp., *Eubacterium rectale*, and *Blautia* spp., are depleted. Additionally, bacteria involved in key metabolic processes, such as mineral metabolism (*Collinsella aerofaciens*, linked to iron metabolism), bile acid metabolism (*Butyricicoccus pullicaecorum*), and the urea cycle (*Bifidobacterium longum*), also show significant reductions (Ning et al., 2023). By deepening our understanding of gut microbiota and metabolites, we can enhance the quality of life for patients with IBD.

## 5 Limitations of the study

This study conducted a systematic search of the WOS database, providing a comprehensive and objective analysis of research progress in the field of metabolites and IBD over the past three decades. It highlights the contributions of authors, countries, research institutions, and journals, and offers a bibliometric analysis to predict future research trends, thereby providing valuable insights for the direction of future studies. However, several limitations should be noted. First, due to constraints in bibliometric software, only literature from the WOS SCIE database was retrieved, excluding potentially relevant studies from other databases, which may have introduced potential bias. Second, the study focused solely on English-language articles, which may have overlooked important non-English research. Third, the analysis included only journal articles and reviews, excluding conference papers and books, which may contain innovative or early-stage findings. Fourth, our data was collected within the first day, so some articles may not have been published at the time of retrieval. This may potentially affect the completeness of our dataset. Finally, as a quantitative method, bibliometric analysis cannot fully assess the quality of the literature reviewed. Therefore, the results should be interpreted in conjunction with in-depth domain knowledge and qualitative appraisal.

## 6 Conclusion

Research on metabolites and IBD has advanced rapidly, with *Inflammatory Bowel Diseases* emerging as the leading journal in the field. Both China and the USA are key contributors, each having three institutions ranked in the top ten. Recent emerging keywords, such as “*gut microbiota*,” “*inflammation*,” and “*pathogenesis*” reflect the growing focus on these areas. Despite substantial progress, several challenges remain. Future studies will continue to explore the relationship between metabolites and IBD, with a particular emphasis on addressing metabolic imbalances in IBD patients.

## Data Availability

The original contributions presented in the study are included in the article/supplementary material, further inquiries can be directed to the corresponding author.
